# Frontiers in fungal phosphatases: molecular diversity, regulatory mechanisms, analytical methodologies, ecological significance, and prospects for sustainable utilization

**DOI:** 10.3389/fbioe.2026.1735288

**Published:** 2026-02-20

**Authors:** Samiyah Saeed Al-Zahrani

**Affiliations:** Department of Biology, Faculty of Science, Al-Baha University, Al-Baha, Saudi Arabia

**Keywords:** biotechnological application, ecological function, fungal phosphatases, molecular regulation, phosphate solubilization, sustainable agriculture

## Abstract

Phosphorus is an indispensable macronutrient essential for all forms of life, as it plays a central role in cellular energy metabolism, nucleic acid synthesis, and structural integrity. Since organisms can only absorb dissolved inorganic phosphate, the phosphatase enzyme is important in the process of converting organic phosphorus into forms that are bioavailable. Fungal phosphatases are a vastly diverse and heterogeneous functional and structural category that catalyzes the liberation of phosphates in a wide variety of organic compounds and facilitates the mobilization of phosphorus in the soil and symbiotic interactions. This review summarizes the existing information on fungal phosphatases, their classification, molecular regulation, methods of their analysis, ecological significance, and biotechnological use. Bibliometric analysis has been conducted using 3,944 publications published between 1944 and June-2025, and the analysis rate has shown an increase of 7.11% which indicates the rising relevance of the research. Phosphate-sensitive transcriptional networks (PHO/PHR pathways), nutrient signaling (TOR), MAPK cascades, and post-translational modifications control their activity. Analytical methods have either the traditional colorimetric assays or fluorometric and omics-based ones, such as transcriptomics and proteomics. These enzymes mediate organic phosphorus mineralization, symbiotic nutrient exchange in mycorrhizal systems, saprotrophic decomposition, and global phosphorus cycling, which are ecologically relevant. Its uses would be in biofertilizers, soil nutrient management, recovery of phosphorus in waste, industrial bioprocesses, and climate-smart agriculture. Nevertheless, a number of gaps exist in terms of the phosphatase diversity in non-model fungi, complexity of regulatory networks, and methodological sophistication. To promote sustainable phosphorus management, the combination of molecular, ecological and applied viewpoints is a requirement, especially due to the global exhaustion of phosphorus resources and the necessity of environmental sustainability.

## 1 Introduction

Phosphorus constitutes a critical element essential for the survival of all living organisms, attributable to its indispensable function in energy transference, nucleic acid synthesis, and as a structural component of cellular architecture (Butusov and Jernelöv, 2013; Margalef et al., 2017). Given that organisms are capable of assimilating only dissolved phosphate, the enzymatic hydrolysis of organic phosphorus compounds is imperative to render this nutrient bioavailable (Cembella et al., 1982). Microbial entities and plant root systems secrete phosphatase enzymes, which facilitate the cleavage of phosphate moieties from organic molecular structures, thereby transforming phosphorus into bioavailable inorganic forms (Zhu et al., 2024). Among these microbial taxa, phosphate-solubilizing fungi, including both ectomycorrhizal and arbuscular mycorrhizal forms, play a pivotal role in phosphorus mineralization and solubilization processes, which collectively exert profound effects on plant growth, seed formation, crop phenological development, and resistance to phytopathogenic diseases (Etesami et al., 2021; Mehta et al., 2019).

Fungal phosphatases represent a heterogeneous assemblage of enzymes that facilitate the hydrolysis of phosphate esters, a process imperative for an array of molecular, ecological, and applied roles (de Assis et al., 2015). This group includes serine/threonine (Ser/Thr) protein phosphatases as well as acid/alkaline phosphomonoesterases, both of which are integral to signal transduction pathways, nutrient recycling, and metabolic regulation in fungi (Winkelstroter et al., 2015b). Whereas phosphatases, classified as a subgroup within esterases, specifically target phosphoric acid esters of alcohols and are systematically categorized into phosphomonoesterases, phosphodiesterases, and polyphosphatases based on the specificity of their substrates. Phosphomonoesterases, alternatively referred to as phosphatic monoester hydrolases, are further differentiated into acid phosphatases and alkaline phosphatases (ALPs), contingent upon their optimal pH activity (Bhalla et al., 2025). Predominantly, ALPs are of microbial derivation, whereas acid phosphatases (AcPases) are secreted by both plant and microbial sources (Rani et al., 2012). Fungal ectophosphatases, frequently identified as membrane-bound AcPases released by ectomycorrhizal fungi, play a critical role in the degradation of organic phosphorus compounds within the soil, facilitating their conversion into inorganic forms that are accessible for plant uptake (Freitas-Mesquita and Meyer-Fernandes, 2014; Ho and Zak, 1979). Within these symbiotic associations, ectophosphatase activity significantly augments plant nutrient absorption, particularly phosphorus, an effect often amplified by the presence of elevated nitrogen levels (Chiu and Paszkowski, 2019). Phosphatases are conventionally classified into five principal categories: alkaline phosphatases, high molecular weight acid phosphatases (HMW AcPases), low molecular weight acid phosphatases (LMW AcPases), purple acid phosphatases (PAPs), and protein phosphatases (PPs) (de Araujo et al., 1976; Li et al., 2021). Among these classifications, acid phosphatase activity is predominantly measured in environmental and biochemical research (Olczak et al., 2003). Although both AcPases and ALPs coexist within soil matrices, contemporary analytical methodologies frequently quantify only one enzyme class or substrate type at a time, predominantly emphasizing monophosphates. This focus persists despite the simultaneous hydrolysis of monoester and diester phosphates (Zheng et al., 2021).

From an applied perspective, fungal phosphatases exhibit significant potential for applications in biotechnology and industry, particularly concerning environmental remediation, biofertilizer development, and sustainable bioprocessing (Fu et al., 2024; Tian et al., 2024). Recent advancements in genetic engineering have facilitated the production of high-activity phosphatases within optimized fungal hosts, resulting in efficient biocatalysts for the treatment of wastewater, the recovery of phosphate from agricultural runoff, and the enhancement of soil fertility without the use of chemical additives (Fazeli-Nasab and Rahmani, 2021). Although many individual reviews have addressed phosphatase biochemistry, mycorrhizal phosphorus cycling, or biotechnological applications in isolation, none have provided the integrative framework necessary to translate molecular-level discoveries into tangible agronomic and environmental outcomes. This review addresses that critical deficiency by offering the first comprehensive synthesis that explicitly connects enzymatic mechanisms with ecological functions and biotechnological potential. In contrast to existing reviews that remain constrained within narrow disciplinary domains, we integrate five interrelated dimensions enzymatic heterogeneity, molecular regulation, methodological standardization, ecological roles, and translational applications that have thus far been treated in a fragmented manner in the literature. By systematically examining persistent knowledge gaps in non-model fungal phosphatases, their environmental responsiveness, the lack of standardized methodological approaches, and their underexplored biotechnological utility, this synthesis advances the conceptualization of fungal phosphatases as central leverage points for addressing global phosphorus sustainability and provides a foundational framework for future research that transcends traditional disciplinary boundaries.

## 2 A bibliometric overview of fungal phosphatase

**FIGURE 1:**
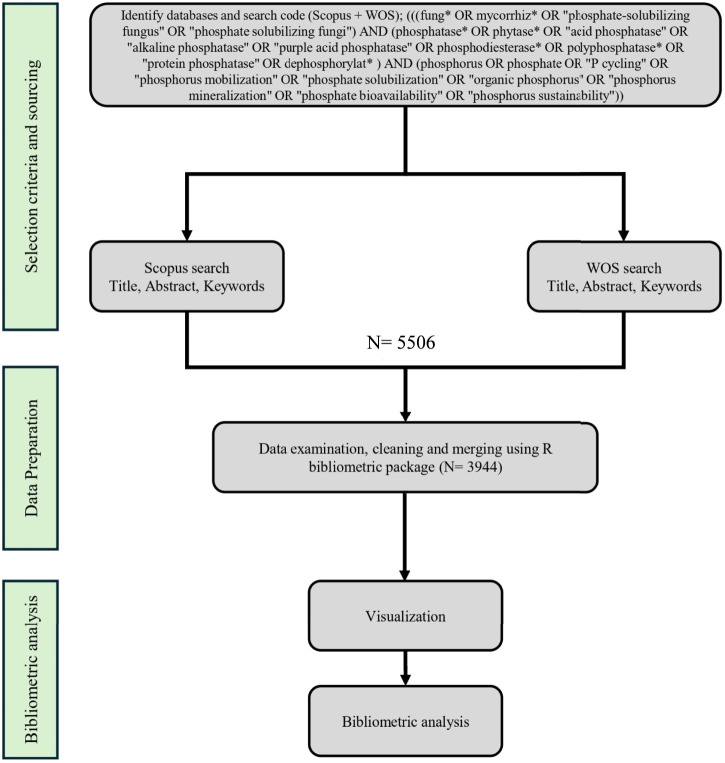
Flowchart illustrating the bibliometric procedures employed in this study. The methodological workflow comprises three principal stages: (1) selection criteria, (2) data preparation, and (3) bibliometric analysis.

**FIGURE 2:**
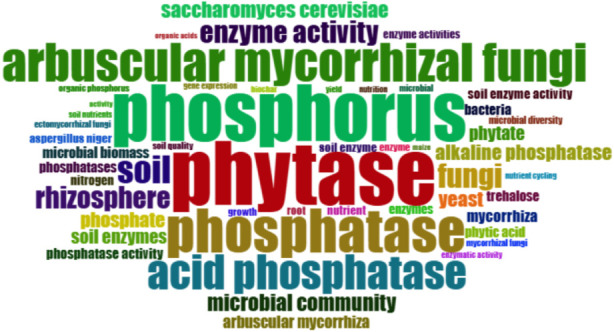
Word cloud illustrating the most frequently occurring terms (frequency >24) in publications related to fungal phosphatases. The visualization was generated using data obtained from Scopus and Web of Science, based on a sample of 3,944 documents, and keyword statistics computed in Biblioshiny.

A bibliometric analysis was conducted as summarized in Figure 1, utilizing specific keyword strings to explore the literature concerning fungal phosphatases. The search query composed of TITLE-ABS-KEY[(fung* OR mycorrhiz* OR “phosphate-solubilizing fungus” OR “phosphate solubilizing fungi”) AND (phosphatase* OR phytase* OR “acid phosphatase” OR “alkaline phosphatase” OR “purple acid phosphatase” OR phosphodiesterase* OR polyphosphatase* OR “protein phosphatase” OR dephosphorylat*] AND (phosphorus OR phosphate OR “P cycling” OR “phosphorus mobilization” OR “phosphate solubilization” OR “organic phosphorus” OR “phosphorus mineralization” OR “phosphate bioavailability” OR “phosphorus sustainability”) yielded 3,233 articles from the Scopus database and 2,273 articles from the Web of Science (WOS). After merging metadata from both databases, the consolidated dataset comprised 3,944 documents, reduced from an original size of 5,506 documents due to the elimination of 1,562 duplicates. The bibliometric analysis encompassed publications from 1944 to 2025 and included 1,083 distinct sources (journals, conference proceedings, and monographs), indicating a robust and sustained expansion of research activity in this area. An annual growth rate of 7.11% demonstrates a consistent increase in scholarly output and academic interest in this domain over the past 8 decades. The mean document age of 13.8 years suggests a mature yet continuously evolving field, wherein both seminal and recent contributions exert substantial influence. Scholarly impact is reflected by an average of 40.21 citations per document, evidencing considerable academic visibility and interdisciplinary connectivity across domains such as microbiology, soil science, and environmental biotechnology. The identification of 17,384 Keywords Plus and 8,585 Author Keywords indicates pronounced thematic heterogeneity and multidimensional research orientations, spanning molecular-scale investigations to applied studies, as illustrated in Figure 2. Authorship patterns further delineate the structure of the research community. A total of 11,595 authors have contributed to this body of literature, with only 137 authors responsible for single-authored documents. In absolute terms, single-authored publications number 156, representing less than 4% of the total output. These emphasize the predominance of collaborative research constellations and the centrality of co-authorship networks. The mean number of co-authors per article is 5.24, reinforcing the view that research on fungal phosphatases is intrinsically collaborative and integrative, frequently intersecting with microbiology, agronomy, ecology, and biochemistry. The international co-authorship rate of 16.24% indicates a moderate level of cross-national collaboration. This metric suggests an expanding, though not yet fully optimized, landscape of international research partnerships, likely driven by common global concerns related to phosphorus sustainability. At the same time, it highlights a substantial untapped potential for intensifying transnational networking, capacity building, and knowledge exchange. These patterns are broadly consistent with those documented in other emergent biotechnological domains that rely on interdisciplinary expertise and shared research infrastructure.

## 3 Classification and diversity of fungal phosphatases

**FIGURE 3:**
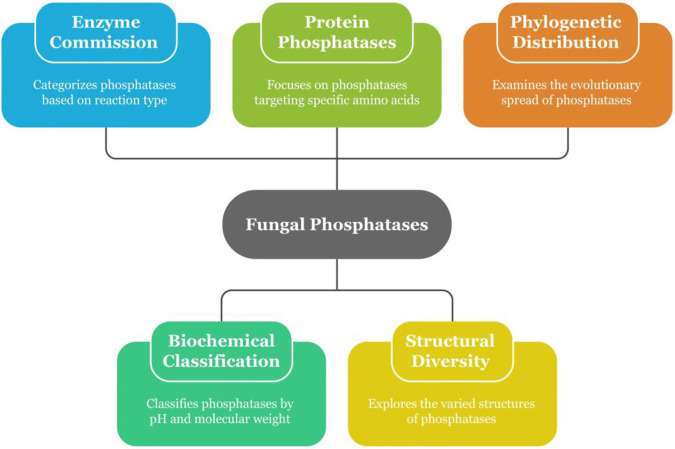
Overview of fungal phosphatases classifications.

Fungal phosphatases demonstrate significant structural and functional heterogeneity, indicative of their participation in a multitude of physiological, biochemical, and ecological functions (Chen et al., 2024). These enzymes are systematically categorized according to their catalytic attributes, substrate specificity, and molecular features (Millan, 2006). Classification methodologies, as depicted in Figure 3, such as the Enzyme Commission (EC) framework and biochemical classifications, offer systems for distinguishing predominant phosphatase categories, including phosphomonoesterases, phosphodiesterases, and protein phosphatases. This heterogeneity encompasses variations in optimal pH, metal ion dependencies, molecular mass, and catalytic mechanisms, which collectively underpin enzyme function and ecological roles.

The EC classification system systematically categorizes hydrolases that act on ester bonds into distinct subgroups based on the type of bond cleaved and the chemical properties of the substrates involved (McDonald et al., 2015). Within this classification scheme, fungal phosphatases are classified among esterases that specifically hydrolyze phosphoric acid esters of alcohols, and they are allocated to three primary EC classes: phosphomonoesterases (EC 3.1.3), phosphodiesterases (EC 3.1.4), and other phosphatases/related hydrolases (Leake and Miles, 1996). Leake and Miles (Leake and Miles, 1996) provided direct evidence of extracellular phosphodiesterase production by an ericoid mycorrhizal fungus when utilizing DNA as a phosphorus source, thereby demonstrating the functional expression of EC 3.1.4 activities by fungal taxa in natural environments. Various studies on organophosphate esterases, including monoesterases (alkaline phosphatases) and triesterases in microorganisms, underscore the ecological significance and diversity of phosphatase reactions categorized under the EC 3.1. designation (Tehara and Keasling, 2003). Whereas, phosphomonoesterases (EC 3.1.3) facilitate the hydrolysis of phosphomonoesters, resulting in the production of inorganic phosphate and an alcohol (Kuznetsova et al., 2005). In fungal systems, these enzymatic activities manifest as both extracellular and intracellular enzymes and are conventionally classified as acid phosphatases and alkaline phosphatases (ALPs), distinguished by their pH optima and molecular characteristics (Leake and Miles, 1996). Empirical investigations have documented the activities of fungal acid and alkaline phosphomonoesterases: whereas they detailed that extracellular phosphomonoesterase and phosphodiesterase activities associated with DNA/phosphate utilization by mycorrhizal fungi, illustrating the contribution of fungal phosphomonoesterases to the mobilization of organic phosphorus (Leake and Miles, 1996). Extensive microbiological research on organophosphate esterases emphasizes alkaline phosphatase (a monoesterase) as a predominant enzyme studied for its role in releasing inorganic phosphate under conditions of phosphate scarcity, in alignment with EC 3.1.3 categorization (Ragot, 2016; Tehara and Keasling, 2003). The bifurcation into acid versus alkaline phosphatases is indicative of observable differences in pH optima and ecological distribution: fungi inhabiting acidic environments typically exhibit acid phosphatase activity, whereas those in more alkaline habitats may express ALPs, a trend corroborated by analyses of fungal phosphatase activity in environmental and culture-based studies (Della Monica et al., 2018). While, phosphodiesterases (EC 3.1.4) are enzymes that catalyze the hydrolysis of phosphodiester bonds within nucleic acids, phospholipids, or cyclic nucleotides (Morton, 1965). The activities of fungal phosphodiesterases have been directly observed; Leake and Miles (Leake and Miles, 1996) identified both extracellular and cell-wall-associated phosphodiesterase produced by *Hymenoscyphus ericae*, which facilitated growth on DNA as the exclusive phosphorus source. Further biochemical analyses of fungal phosphodiesterase fractions, such as those from *Aspergillus niger*, reveal heterogeneous diesterase activities with varying substrate specificities, indicating that fungal EC 3.1.4 enzymes exhibit significant biochemical diversity (Jarosch, 2016). Additionally, multiple studies have demonstrated overlapping or dual activities among some microbial phosphodiesterases, challenging strict EC classification but highlighting the true diversity of these enzymes (Garner et al., 2024). Such dual functionality has been documented both in non-fungal microorganisms and within fungal systems, suggesting that fungal phosphodiesterases may similarly exhibit broad substrate specificity and play crucial roles in the recycling of nucleic acid-derived phosphorus (Tehara and Keasling, 2003). In addition, fungi exhibit a range of phosphatase activities and associated hydrolases that are integral to phosphate metabolism and their ecological adaptability. Furthermore, multifunctional hydrolases that integrate diesterase, phosphatase, and nucleotidase functions are also present. Recent advancements in the identification of microbial enzymes in bacteria highlight the biochemical diversity of these enzymes and suggest the presence of similar capabilities or functional analogs in fungal genomes (Freeman et al., 2025). Studies on fungal pathogens have shown that disruption of polyphosphate utilization affects phosphate homeostasis within cells and influences virulence, thereby underscoring the biological significance of polyphosphatase activity in fungi (Zyla et al., 1995). Additionally, enzymes capable of hydrolyzing organophosphates, such as triesterases and phosphotriesterases, are being explored within microbial systems for applications in environmental detoxification and nutrient recycling (Tehara and Keasling, 2003).

The research delineates the biochemical classification of fungal phosphatases, focusing on ALPs, AcPases, and PAPs, with distinctions made according to molecular weight. ALPs are classified as phosphomonoesterases that exhibit optimal catalytic function at alkaline pH levels, facilitating the release of inorganic phosphate from an extensive array of monoester substrates (Saavedra and Baltar, 2025). The biochemical identification and characterization of fungal ALP activities have been documented through both environmental and laboratory investigations. For instance, the study by Della Monica et al. (2018), highlights the presence of alkaline phosphatase activity across various fungal strains and observes that these alkaline activities can coexist with acid phosphatase activities, albeit frequently exhibiting lower activity compared to acid phosphatases under numerous tested conditions, this due to differences in optimal pH ranges, substrate specificity, and regulatory control mechanisms as ALPs are most active under alkaline conditions, which are less common in many natural soils and rhizosphere environments, whereas AcPases function efficiently under the acidic conditions typical of most soils. Furthermore, the biochemical profiling of ectophosphatase activities in *Candida albicans* reveals an alkaline, Cu^2+^ dependent ectophosphatase, which is stimulated by Mg^2+^, setting it apart from an acidic, Cu^2+^ independent activity, thereby providing functional evidence for distinct ALP-like activities at the fungal cell surface (Freitas-Mesquita et al., 2025). The co-regulation by divalent metal ions and responsiveness to classical phosphatase inhibitors are consistent biochemical characteristics of these fungal alkaline activities, as noted in targeted ectophosphatase characterizations (Freitas-Mesquita et al., 2025). Additionally, alkaline phytases, which are a functionally related group, hydrolyze phytate most effectively at higher pH levels and have been biochemically differentiated from histidine-acid phytases in comparative analyses (Oh et al., 2004). While acid phosphatases (AcPases) are a class of phosphomonoesterases that exhibit optimal activity under acidic pH conditions and are ubiquitously present among fungi, playing a crucial role in the mineralization of organic phosphorus within acidic environments (Oliveira et al., 2025). Empirical evidence indicates that the activity of fungal acid phosphatases frequently surpasses that of alkaline phosphatases across a wide array of fungal species and environmental conditions. This pattern is corroborated by the findings of Della Monica et al. (2018), who reported consistently higher acid phosphatase activity compared to alkaline activity across various pH treatments and time points in culture assays. Cell-wall-associated acid phosphatases, such as those found in *Aspergillus fumigatus* (PhoAp), have been subject to molecular and biochemical characterization (Freitas-Mesquita and Meyer-Fernandes, 2014). PhoAp is identified as a glycosylphosphatidylinositol-anchored glycoprotein with a molecular weight of approximately 80 kDa, demonstrating enzymatic activity on both phosphate monoesters and diesters and subject to repression by phosphate availability, thereby illustrating typical regulatory and biochemical characteristics of fungal AcPases (Bernard et al., 2002). Historical and ecological investigations of ericoid mycorrhizal fungi similarly highlight the role of extracellular acid phosphatases in mobilizing organic phosphorus sources to support fungal growth (Lemoine et al., 1992). Studies focusing on ectophosphatases have concluded that acid phosphatase activities generally exhibit insensitivity to certain divalent metal ions, such as Mg^2+^, which typically enhance alkaline phosphatase activities, thereby further distinguishing these biochemical classes (Freitas-Mesquita et al., 2025). Moreover, purple acid phosphatases (PAPs) are a category of metallophosphatases, distinguished by a distinctive purple hue that results from their binuclear metal center, typically comprising iron-zinc or iron-manganese complexes (Schenk et al., 2008). These enzymes have been extensively characterized in both plant and bacterial systems. Current research is increasingly identifying fungal sequences related to acid phosphatase families, inclusive of PAPs, through comprehensive sequence curation and phylogenetic analyses. A recent curated phylogenetic study focusing on fungal acid phosphatase and phytase sequences has delineated several distinct clades, which encompass groups corresponding to classical acid phosphatases, phytases, and sequences annotated as PAPs (Gontia-Mishra and Tiwari, 2013). This suggests that fungal genomes encode enzymes homologous to PAPs and that these enzymes represent a discernible biochemical and phylogenetic subset within the broader category of fungal acid phosphatases (Gomez-Gallego et al., 2025). Investigations centered on plant PAPs have established specific sequence motifs and catalytic characteristics that facilitate the identification of PAP homologues in non-plant genomes, thereby substantiating the detection of fungal PAPs through bioinformatic profiling (Schenk et al., 2000).

**FIGURE 4:**
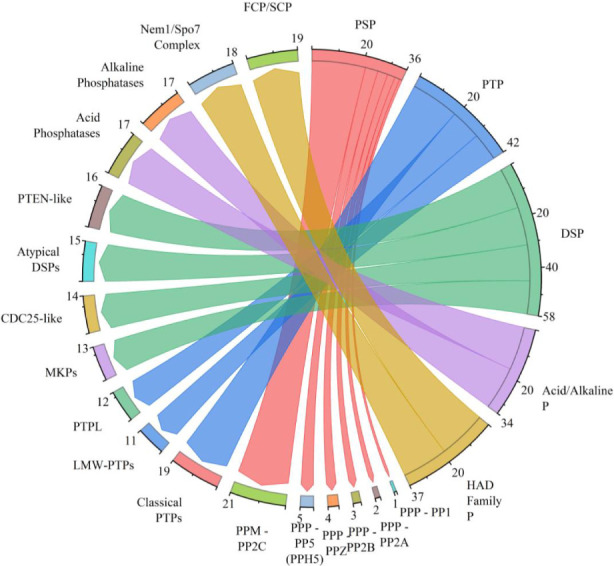
Classification of fungal phosphatases, providing an overview of the major classes and their corresponding representative families and subfamilies, the graph was generated using OriginPro, version 2024.

Serine/threonine protein phosphatases represent several conserved families, including the PPP family members such as PP2A, PP2B (calcineurin), and PP1, as well as PPM/PP2C family members, which are also detected in fungi, as depicted in Figure 4 (Chen et al., 2016; Yang and Arrizabalaga, 2017). These phosphatases play pivotal roles in processes such as signal transduction, stress response, morphogenesis, and virulence (Ortiz-Urquiza and Keyhani, 2015). Calcineurin, also known as protein phosphatase 2B (PP2B), serves as a prototypical example of a fungal Ser/Thr PP, it functions as a heterodimer, consisting of a catalytic A subunit and a regulatory B subunit, and is activated by calcium and calmodulin, with its sequence and quaternary structure being conserved across eukaryotic organisms (Fox and Heitman, 2005; Rusnak and Mertz, 2000). Genetic and biochemical disruptions of calcineurin lead to impairments in thermotolerance, hyphal growth, and virulence in a range of fungal pathogens, including *Cryptococcus neoformans*, *Candida albicans*, and *Aspergillus fumigatus*, this underscores the crucial role of calcineurin in fungal physiology and pathogenicity (Bader et al., 2003; Fox et al., 2001; Odom et al., 1997). For example, mutants lacking the calcineurin A and B subunits fail to grow at host temperatures and exhibit a virulence in *C. neoformans*, highlighting its indispensability for infection-related attributes (Odom et al., 1997; Rasmussen et al., 1994). Furthermore, loss or pharmacological inhibition of calcineurin in *Candida* species increases cellular sensitivity to antifungal agents and diminishes virulence in animal models (Brown et al., 2013; Yu et al., 2014). Beyond calcineurin, other Ser/Thr phosphatases regulate nutrient-sensing and developmental programs. TOR pathway–regulated PP2A and PP2C phosphatases modulate responses to nutrient availability and carbon source, and their activities change under rapamycin or nutrient deprivation, linking Ser/Thr dephosphorylation to metabolic and developmental control (Brown et al., 2013). In *Aspergillus* and other filamentous fungi, multiple Ser/Thr phosphatases contribute to germination, hydrolytic enzyme production, and primary metabolism; mutations in these phosphatases alter growth on cellulose or affect secretion of hydrolytic enzymes, underscoring their biochemical integration with fungal ecology and biotechnology-relevant processes (de Assis et al., 2015). Protein tyrosine phosphatases, encompassing both classical and low-molecular-weight PTPs, are integral components of fungal genomes, where they have been associated with specialized regulatory functions, notably in secondary metabolism and developmental processes (Yu and Zhang, 2018). Filamentous fungi are known to encode a diverse array of PTP family members, including both dual-specificity and classical PTPs (Arino et al., 2011). Comparative genomic analyses, such as those conducted in *Aspergillus fumigatus*, have identified a substantial repertoire of PTP genes, underscoring the significance of tyrosine dephosphorylation as a crucial facet of the fungal phosphatase repertoire (Winkelstroter et al., 2015a). Empirical data indicate that the inhibition of tyrosine phosphatase activity can modulate secondary metabolite synthesis, exemplified by the impact of PTP inhibitors on aflatoxin production in *Aspergillus flavus*, thereby implicating PTPs in the regulation of biosynthetic pathways with virulence-associated or ecological relevance (Inoguchi et al., 2019). Dual-specificity phosphatases (DUSPs), often referred to as MAP kinase phosphatases (MKPs) in the context of their action on mitogen-activated protein kinases (MAPKs), are enzymes that catalyze the removal of phosphate groups from both phosphoserine/threonine and phosphotyrosine residues (Jeffrey et al., 2007). These enzymes serve as critical regulators of MAPK-mediated signaling pathways in fungi. DUSPs are characterized by a conserved domain architecture featuring family-specific interaction motifs that facilitate substrate recognition and enable regulatory coupling to stress response mechanisms (Huang and Tan, 2012). For instance, yeast proteins such as Sdp1 demonstrate the role of MKPs in linking oxidative or other types of stress to selective inactivation of MAPKs (González-Rubio et al., 2019). In fungal systems, MKPs are instrumental in modulating MAPK pathways responsible for maintaining cell wall integrity, osmotic balance, and developmental processes (Gonzalez-Rubio et al., 2019). Genomic and functional analyses have identified fungal DUSPs that exert influence over these pathways, thus establishing a connection between dual-specificity dephosphorylation processes and adaptive responses as well as pathogenic characteristics (Gonzalez-Rubio et al., 2019).

Fungal protein phosphatases exhibit a remarkable structural diversity that is fundamental to their catalytic mechanisms and regulatory capabilities. Calcineurin (PP2B), a notable example, contains a dinuclear metal center at its active site and necessitates calcium ions and calmodulin for its activation. The conserved architecture of calcineurin accounts for its susceptibility to immunosuppressive complexes, such as FK506–FKBP12 or cyclosporin A (CsA) cyclophilin, which inhibit phosphatase activity by stabilizing inhibitory ternary complexes (Fox and Heitman, 2005; Rusnak and Mertz, 2000). This structural foundation has been leveraged to design fungal-selective calcineurin inhibitors, specifically modified FK506/FK520 analogs, which minimize immunosuppression while maintaining antifungal effectiveness. This demonstrates the potential of detailed structural insights into phosphatase-inhibitor interactions to facilitate the development of targeted therapeutic strategies (Hoy et al., 2022; Rivera et al., 2023). Ser/Thr phosphatase families such as PP2A, PP1, and PP2C exhibit variations in their catalytic metal requirements, regulatory subunits, and structural configurations. Specifically, PP2C phosphatases, classified as type 2C, belong to the PPM enzyme family and are characterized by their single-polypeptide structure and reliance on metal cofactors. These enzymes achieve substrate specificity not through stable regulatory subunits but by expressing multiple isoforms (Arino et al., 2011). Furthermore, low molecular weight protein tyrosine phosphatases and dual-specificity phosphatases are identified by distinct active-site motifs and substrate binding domains, which determine their affinity for phosphotyrosine, phosphoserine, and/or phosphothreonine residues. Structural and biochemical analyses across diverse eukaryotic systems have provided foundational models for elucidating the enzymatic mechanisms of fungal phosphatases (Gonzalez-Rubio et al., 2019; Lee et al., 2020). The interplay between conserved catalytic cores and adaptable regulatory modules accounts for the diverse cellular localizations, such as cytosolic, cell-wall/ectophosphatases, and membrane-associated and specialized functional roles observed in fungal species (Freitas-Mesquita et al., 2025).

## 4 Molecular regulation of fungal phosphatases

The availability of phosphate serves as a predominant transcriptional signal that regulates phosphatase expression within eukaryotic organisms (Azevedo and Saiardi, 2017). Comparative genomic analyses have revealed the conservation of PHO/PHR-like components in both fungi and their symbiotic counterparts. Genomic assessments of arbuscular mycorrhizal fungi have reported the preservation of PHO signaling components, in conjunction with the TOR, cAMP-PKA, and SNF1 pathways (Zhou et al., 2021). This suggests the existence of a phosphate-responsive regulatory module capable of modulating phosphatase gene expression in AM fungi, and by extension, other fungal taxa (Zhou et al., 2021). Studies on phosphorus starvation in plants and fungi indicate that the genes encoding purple acid phosphatase and acid phosphatase are transcriptionally activated under conditions of Pi limitation, thereby demonstrating a conserved Pi-responsive regulatory mechanism of phosphatase expression across biological kingdoms (Huong To et al., 2022; Hurley et al., 2010). Furthermore, sequence-based analyses of fungal acid phosphatases have identified Pi-repressible enzymes, such as PhoAp in *Aspergillus fumigatus*, that are inhibited by the presence of phosphate and activated during Pi deprivation, offering direct fungal instances of PHO-like regulation of phosphatase genes (Bernard et al., 2002). While the mediation of PHO/PHR responses by specific transcription factors has been well-documented, in plants, regulators belonging to the PHR family, such as PHR2, have been shown to directly regulate PAP gene expression under conditions of Pi deprivation (Huong To et al., 2022). This mechanism is paralleled by orthologous systems, wherein transcription factors upregulate Pi-scavenging enzymes as part of the eukaryotic phosphate starvation responses (Wang et al., 2003). While the transcriptional machinery involved in PHO responses, particularly Pho4 in *Saccharomyces*, is thoroughly characterized, comparative studies in arbuscular mycorrhizal fungi and filamentous fungi reveal conserved sets of nutrient-responsive regulators. These regulators are postulated to perform analogous functions in the regulation of phosphatase gene expression (Zhou et al., 2021). As an empirical example, the phosphate-repressible transcriptional regulation of PhoAp in *Aspergillus fumigatus* illustrates the transcription factor-mediated regulation of fungal acid phosphatase genes under Pi-deprivation conditions (Bernard et al., 2002).

Protein phosphatases are intricately regulated through phosphorylation networks and interactions with kinase signaling pathways. Nutrient signaling via the Target of Rapamycin (TOR) pathway exerts control over the PP2A/PP2C family of phosphatases. Specifically, the inhibition of TOR, achieved through agents such as rapamycin or nutrient deprivation, modulates the interaction of regulatory factors, such as Tap42, with PP2A-type phosphatases. This modulation results in the swift activation or altered targeting of PP2A and PP2A-like enzymes, thereby linking nutrient availability to phosphatase activity and subsequent transcriptional responses (Cutler et al., 2001; Wang et al., 2003). Dual-specificity phosphatases (MKPs) are upregulated in response to stress, functioning to dephosphorylate Mitogen-Activated Protein Kinases (MAPKs). In the yeast model, the stress-inducible MKP Sdp1 mediates the downregulation of Slt2 MAPK phosphorylation following heat or oxidative stress, illustrating the role of phosphatase induction and activity in terminating kinase signaling during adaptive responses (Hahn and Thiele, 2002).

Extracellular and cell wall-associated fungal phosphatases frequently necessitate processing through the secretory pathway and subsequent glycosylation to achieve appropriate localization and enzymatic activity. Specifically, the *Aspergillus fumigatus* cell wall acid phosphatase, PhoAp, is characterized as a glycoprotein possessing a glycosylphosphatidylinositol (GPI) anchor. The glycosylation and processing of this anchor are pivotal in determining its association with the cell wall or its release into the culture filtrate, a process essential to its function as an ectophosphatase involved in mobilizing organic phosphorus (Bernard et al., 2002). More generally, phosphatidic acid phosphatases and secreted acid phosphatases in eukaryotic organisms undergo post-translational modifications that significantly impact their stability, secretion, and extracellular functionality. This is consistent with the observed reliance of extracellular phosphatase activity on processes of maturation and trafficking (Gomez-Gallego et al., 2025; Hurley et al., 2010). The assembly of multimeric complexes involving regulatory subunits and specific protein-protein interfaces constitutes the primary mechanism by which highly conserved catalytic phosphatase cores achieve substrate specificity, precise localization, and regulated activity. Type-1 phosphatases (PP1) rely on an array of regulatory subunits that dock through conserved motifs such as RVXF to direct the catalytic subunit PP1c to specific substrates and cellular compartments (Gibbons et al., 2005). Similarly, fungal PP1 systems, including the Glc7 and PPZ families, exhibit specialized regulatory partners across different species, with structural variations, such as the N-terminal extensions observed in PPZ, contributing to distinct regulatory mechanisms (Casamayor et al., 2022). Phosphatases of the PP2A family are organized into heterotrimeric holoenzymes, wherein B-type regulatory subunits, including the B56/PR61 classes, dictate substrate selection and biological outcomes. In fungi and fungal pathogens, the PP2A regulatory subunit B56 (MoB56) and the atypical catalytic subunit Ppg1 form complexes that are crucial for growth and pathogenicity in *Magnaporthe oryzae*, highlighting the biological significance of specific regulatory subunit-catalytic subunit pairings (Shomin-Levi and Yarden, 2017; Wang et al., 2003). Proteins of the Tap42 (yeast)/α4 family associate with PP2A/PP4/PP6-related catalytic subunits, thereby linking nutrient/TOR signaling to phosphatase regulation. These Tap42–phosphatase interactions are essential for numerous TOR-regulated developmental responses and Sit4/PP2A-type functions in fungi (Cutler et al., 2001; Wang et al., 2003). Collectively, these findings underscore that assembly with regulatory subunits, along with modulators such as Hal3/Cab3 involved in PPZ regulation, and the presence of multifunctional regulatory proteins, are fundamental to the specificity and regulation of fungal phosphatases (Casamayor et al., 2022).

Phosphatases are integral components and effectors within conserved nutrient and stress signaling pathways. The TOR signaling pathway directly modulates the activity of protein PP2A and PP2C, thereby influencing cellular decisions regarding growth, autophagy, and differentiation in response to nutrient availability. The activation of PP2A after TOR inhibition is instrumental in mediating extensive metabolic reprogramming (Cutler et al., 2001; Wang et al., 2003). Calcineurin (PP2B), a calcium and calmodulin-activated serine/threonine phosphatase, is essential for thermotolerance, ion homeostasis, hyphal growth, and pathogenicity in various fungal species, effectively converting Ca^2+^ signals into appropriate stress-adaptive responses (Bader et al., 2003; Odom et al., 1997; Rusnak and Mertz, 2000). Dual-specificity phosphatases modulate MAPK pathways that respond to osmotic, cell wall, and oxidative stressors by terminating MAPK activation to restore homeostasis (Hahn and Thiele, 2002). These instances underscore the pivotal role of phosphatases in sensing nutrient and stress conditions and orchestrating suitable cellular responses. The regulation of phosphatases is intricately connected with the metabolic pathways of carbon, nitrogen, and lipids. In the context of filamentous fungi, Serine/Threonine phosphatases are crucial in modulating germination and primary metabolic processes in response to carbon sources, such as the involvement of PP2A/PP4 in carbon catabolite repression/carbon catabolite derepression (CCR/CCDR). Recent research highlights the role of PP4 complexes in governing carbon catabolite repression mechanisms in the fungus *Magnaporthe oryzae*, thereby illustrating the direct association between phosphatase activity and carbon utilization pathways (Huang et al., 2025). Furthermore, PP2A regulatory modules, such as B56, interact with kinases like NDR kinases, including COT1, to regulate polar growth, branching, and development, effectively linking phosphatase function with both morphogenetic and metabolic states (Shomin-Levi and Yarden, 2017). Within symbiotic and rhizosphere environments, fungal phosphatases collaborate with host transporters and microbial communities to facilitate the mobilization of organic phosphorus, thus integrating phosphatase activity into ecosystem-scale nutrient cycling (Cao et al., 2022; Ren et al., 2018). Additionally, the mobilization of polyphosphate and the action of polyphosphatases impact phosphate homeostasis and the expression of virulence phenotypes in pathogenic organisms, further exemplifying the complex metabolic interactions involved (Ahmed et al., 2022).

The complexity of fungus phosphatase regulating mechanisms relates to the combination of the external phosphate availability, global nutrient conditions, cellular metabolic conditions, and various stress inputs, each of which is signaled by partially different but overlapping cells of molecular circuits (Martin, 2023). Two main regulatory centers in these include the phosphate-responsive phosphatase regulator (involving the PHO regulator) and the nutrient-sensing TOR in the regulation of phosphatase gene transcription and enzyme action under phosphate scarcity (Rouached et al., 2010; Zhou et al., 2021). Comparative genomic studies of arbuscular mycorrhizal fungi show that the common phosphate starvation signaling components are conserved with TOR, cAMP-PKA, and SNF1 pathways, along with the view that a generalized signaling architecture exists that interacts phosphate starvation signals with the cellular energy and nutritional status of the cell (Zhou et al., 2021). In this allosteric mechanism, the dissociation of the Tap42 adaptor protein of PP2A and PP2C phosphatases is stimulated by TOR inactivation, which occurs, among other stimuli, in response to amino-acid or nitrogen deprivation (Santhanam et al., 2004). Whereas the resulting actin release and activation of these phosphatases promotes PHO regulon induction, acting by dephosphorylating and hence activating transcription factors, including Pho4, or functional equivalents PHR orthologs (Lamarche et al., 2008). Furthermore, the TOR Tap42 -PP2A axis is described as a molecular coincidence detector and consolidates signaling of convergent nutrient starvation and, therefore, phosphatase gene expression not only robustly reacts to a combination of nutrient stress and phosphate limitation (Beck and Hall, 1999; Bonneau et al., 2013). Such dual-input control logic will probably be especially useful in oligotrophic settings, in which co-limitation by phosphate and nitrogen occurs. In addition to being involved in nutrient sensing, environmental stresses such as osmotic perturbation, oxidative stress, and pathogen challenge activate mitogen-activated protein kinase (MAPK) cascades that cross with PHO and TOR signaling through common phosphatase effectors (Mohammadi et al., 2021; Zhang et al., 2021). Appropriate examples of these nodes are dual-specificity phosphatases (DUSPs) and MAPK phosphatases (MKPs), such as the enzymes Sdp1 and Ptc1 in yeast, and these are the points that integrate stress-derived with nutrient-derived inputs (Gonzalez-Rubio et al., 2019). While HOG1 MAPK is hyperphosphorylated and inactivated by a combination of dephosphorylation by Ptc1, one of the type 2C phosphatases, PTP2C, and the partially overlapping protein-tyrosine phosphatases, PTP2 and PTP3 (Tatebayashi and Saito, 2023). It is significant to note that the same PP2C phosphatases, which are triggered on TOR inhibition when nutrient-deprived cells are exposed to nutrient deprivation, also catalyze the end of MAPK signaling when cells are exposed to acute stress; therefore, the nutrient-limited conditions can precondition cells in a functional linkage where cells can respond to stress forcefully or abnormally in response to nutrient deprivation. In filamentous and hyaline fungi like *Aspergillus* and *Magnaporthe*, the same principle of integrating rise and fall signals is demonstrated by PP2A -PP4 phosphatase complexes to give rise to both carbon catabolite repression and osmotic stress responses (Anjago et al., 2018; Franck et al., 2015). Such observations confirm a paradigm that phosphatase assemblies can be considered multivalent information-processing centers and not simplistic and single input effectors. This regulatory network is further extended by the cAMP-protein kinase A (PKA) and SNF1/AMPK interactive pathways linked to glucose and general availability of carbon and cellular energy depletion respectively, respectively, and instruct the activity of phosphatases to participating metabolic choices (Nicastro, 2015). In the context of phosphate homeostasis particularly, cAMP- PKA signaling governs secretion of hydrolytic enzymes such as extracellular phosphatases during the condition of carbon starvation resulting in the linkage of carbon status to phosphate uptake (Perez-Diaz et al., 2023; Steyfkens et al., 2018). Simultaneously, SNF1-regulated phosphorylation cascades adjust the activity status of catabolic pathways, such as that of the mineralization of organic phosphorus (Nicastro, 2015). A joint response of cAMP-PKA, SNF1, and TOR in a common group of regulatory phosphatases-primarily PP2A, PP2C, and their proximate effectors- indicate that fungus cells utilize a hierarchical state of decision-making processes where diverse nutrient (phosphate, nitrogen, carbon, energy) and stress signals are collectively integrated to produce more coordinated physiological responses.

## 5 The predominant pathway in which fungal phosphatase plays a pivotal role

The initial focus is on the role of Protein Tyrosine Phosphatase (PTC) Genes in the Mitogen-Activated Protein Kinase (MAPK) signaling pathways within *Saccharomyces cerevisiae*. An Overview of the Four MAPK Pathways in Yeast reveals that *Saccharomyces cerevisiae* encompasses four unique MAPK signaling pathways, each mediating cellular responses to specific environmental cues: the Pheromone Response Pathway (Fus3/Kss1 MAPKs), which orchestrates the mating response to peptide pheromones; the Filamentous Growth Pathway (Kss1 MAPK), which is activated under conditions of nutrient scarcity and promotes invasive growth; the High Osmolarity Glycerol (HOG) Pathway (Hog1 MAPK), which is triggered by osmotic stress to maintain cellular homeostasis; and the Cell Wall Integrity (CWI) Pathway (Slt2/Mpk1 MAPK), which is essential for responding to cell wall perturbations and stress. Each of these pathways adheres to a conserved three-tier kinase module: MAPKKK → MAPKK → MAPK. However, the precise modulation of these pathways by phosphatases is paramount to avert hyperactivation and subsequent cellular dysfunction (González-Rubio et al., 2023; Tatjer et al., 2016). In the context of identifying and classifying PTC Genes within the KEGG MAPK Pathway, analysis has identified many genes, as depicted in (Table 1), which function as phosphatases involved in the regulation of MAPK pathways:

**TABLE 1:** Phosphatase genes regulating MAPK signaling pathways in *Saccharomyces cerevisiae*.

ORF/Gene ID	Gene name	Phosphatase type	MAPK target(s)
YMR036C	MIH1	Tyr-specific PTP (CDC25-like)	Cdc28p (G2/M control)
YNL053W	MSG5	Dual-Specificity Phosphatase	Fus3, Slt2 MAPKs
YOR208W	PTP2	Tyr-specific PTP	Hog1 MAPK
YIL113W	SDP1	Stress-inducible dual-specificity phosphatase	MAPK pathways
YER075C	PTP3	Tyr-specific PTP	Hog1 MAPK
YDL006W	PTC1	Type 2C PP2C Ser/Thr phosphatase	Hog1, Slt2 MAPKs

**FIGURE 5:**
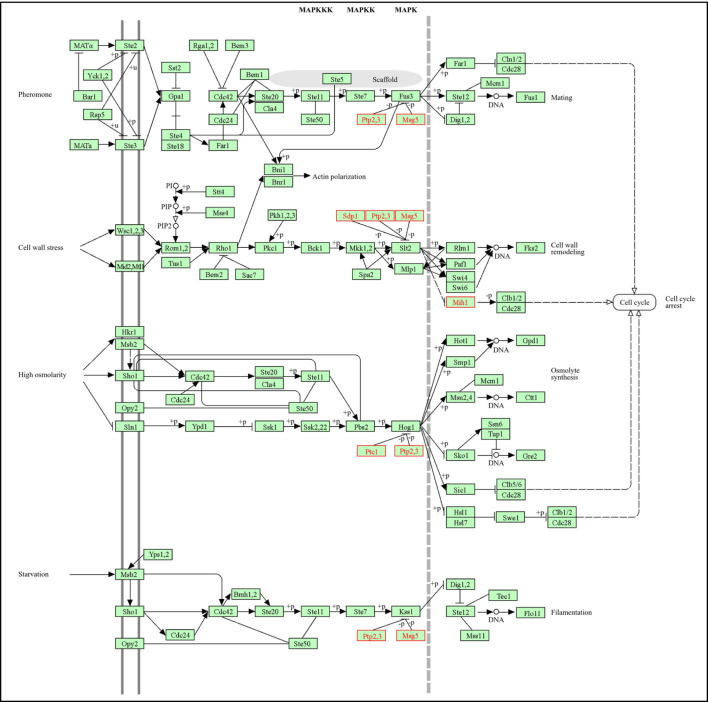
Illustrate the KEGG map of the MAPK signaling pathway in *Saccharomyces cerevisiae* as depicted by Kanehisa and Goto (2000). The map highlights the positions and targets of key phosphatases (ptc1, ptp2, ptp3, msg5, mih1, sdp1) within the mapk cascades, illustrating their roles in regulating hog1, fus3, and slt2 mapks and maintaining signaling fidelity.

The comprehensive functional characterization of PTC/phosphatase genes in *Saccharomyces cerevisiae* highlights their essential regulatory roles within the mitogen-activated protein kinase signaling cascades, as illustrated in (Figure 5). Among these, YDL006W (PTC1) is classified as a Type 2C protein phosphatase, belonging to the magnesium-dependent serine/threonine phosphatase family (PPM family, PP2C) (Warmka et al., 2001). This enzyme plays a pivotal role in the MAPK signaling network, primarily targeting Hog1 MAPK within the High Osmolarity Glycerol (HOG) pathway, which mediates osmotic stress responses. Moreover, Ptc1 also acts on Slt2 MAPK in the Cell Wall Integrity (CWI) pathway (Tatjer et al., 2016), and has broader roles in SNF1/AMPK-mediated glucose regulation (Ruiz et al., 2013). Mechanistically, Ptc1 catalyzes the dephosphorylation of both activating tyrosine and threonine residues on Hog1, thereby terminating the osmotic stress response. In the absence of Ptc1, Hog1 remains hyperphosphorylated, leading to excessive MAPK signaling, disruption of the G2/M cell cycle checkpoint, impaired mitochondrial inheritance, and defective daughter cell separation under heat stress (Gonzalez-Rubio et al., 2023; Warmka et al., 2001). Furthermore, YER075C (PTP3) encodes a tyrosine-protein phosphatase that functions as a phosphotyrosine-specific classical PTP. Its primary target is also Hog1 MAPK within the HOG pathway, where it acts redundantly with PTP2 (YOR208W) to mediate Hog1 inactivation. PTP3 localizes to the cytoplasm and functions as part of a dual-component negative regulatory system with PTP2, dephosphorylating the activating tyrosine residues of Hog1 to maintain the kinase in a hypophosphorylated, inactive state under normal conditions. Additionally, both PTP2 and PTP3 are transcriptionally induced under osmotic stress, ensuring the timely deactivation of the HOG pathway (Jacoby et al., 1997). YOR208W (PTP2), a phosphotyrosine-specific classical phosphatase, also targets Hog1 MAPK as its primary substrate and plays a secondary role in calcium signaling through co-regulation with Msg5. Unlike PTP3, PTP2 exhibits both nuclear and cytoplasmic localization, allowing it to fine-tune Hog1 activity in multiple cellular compartments. It dephosphorylates the activating tyrosine residues of Hog1, thereby restricting maximal kinase activation during osmotic stress (Jacoby et al., 1997). In contrast, YNL053W (MSG5) is a dual-specificity phosphatase capable of dephosphorylating both tyrosine and serine/threonine residues. MSG5 primarily targets Fus3 MAPK in the pheromone response pathway and Slt2 MAPK in the CWI pathway (Gonzalez-Rubio et al., 2019). Moreover, it contributes to the maintenance of low basal signaling through the CWI pathway and exists in two isoforms with distinct regulatory dynamics. Mechanistically, MSG5 acts as a rapid feedback inhibitor—deactivating Fus3 following pheromone stimulation and preventing excessive Slt2 activation through dephosphorylation of both activating residues. While YMR036C (MIH1), a CDC25-like phosphatase, is a phosphotyrosine-specific enzyme primarily targeting Cdc28p, the cyclin-dependent kinase that governs mitotic and meiotic progression. While not directly part of the MAPK cascade, MIH1 coordinates cell cycle transitions with MAPK-regulated stress responses. It dephosphorylates the inhibitory Tyr-15 residue on Cdc28p, thereby promoting the G2/M transition and ensuring synchronization between cell cycle progression and stress signaling pathways. Finally, YIL113W (SDP1) encodes a stress-inducible dual-specificity phosphatase with broad substrate specificity across multiple MAPK cascades. SDP1 expression is transcriptionally upregulated under various stress conditions, providing a rapid, inducible negative feedback mechanism to prevent prolonged MAPK activation. Additionally, it complements the constitutively expressed phosphatases such as Ptc1, Msg5, and PTP2/3, ensuring dynamic and stress-responsive regulation of MAPK activity.

Additionally, in *Saccharomyces cerevisiae*, phosphatase genes play a pivotal role in mRNA surveillance pathways, notably through their involvement in the nonsense-mediated decay (NMD) process. This pathway functions as an essential quality control mechanism, systematically eliminating aberrant mRNAs that harbor premature termination codons (PTCs) to prevent the synthesis of truncated proteins that could be deleterious. Furthermore, NMD exerts regulatory influence over approximately 10% of normal endogenous transcripts, thereby facilitating the fine-tuning of gene expression (Chang et al., 2007; Lejeune, 2022). Through KEGG pathway analysis, several phosphatase genes have been identified as integral to mRNA surveillance. These include TPD3, PPH21, PPH22, GLC7, CDC55, SSU72, RTS1, and PPQ1 as presented in (Table 2), each of which contributes distinct regulatory functions within the pathway.

**TABLE 2:** Phosphatase genes involved in the mRNA surveillance pathway in *Saccharomyces cerevisiae.*

ORF/Gene ID	Gene name	Phosphatase type	Role in mRNA surveillance
YAL016W	TPD3	PP2A Regulatory Subunit A	Protein biosynthesis, translation regulation
YDL134C	PPH21	PP2A Catalytic Subunit	Cell cycle, mRNA stability, NMD indirect regulation
YDL188C	PPH22	PP2A Catalytic Subunit	Redundant with Pph21, mRNA quality control
YER133W	GLC7	PP1 (Type 1 Ser/Thr Phosphatase)	Translation termination, NMD regulation
YGL190C	CDC55	PP2A Regulatory Subunit B	Cell cycle checkpoints, indirect NMD regulation
YNL222W	SSU72	CTD Tyr Phosphatase	mRNA 3′-end processing, transcription termination
YOR014W	RTS1	PP2A Regulatory Subunit B′	Alternative PP2A regulatory subunit
YPL179W	PPQ1	PP1-related phosphatase	Protein quality control

**FIGURE 6:**
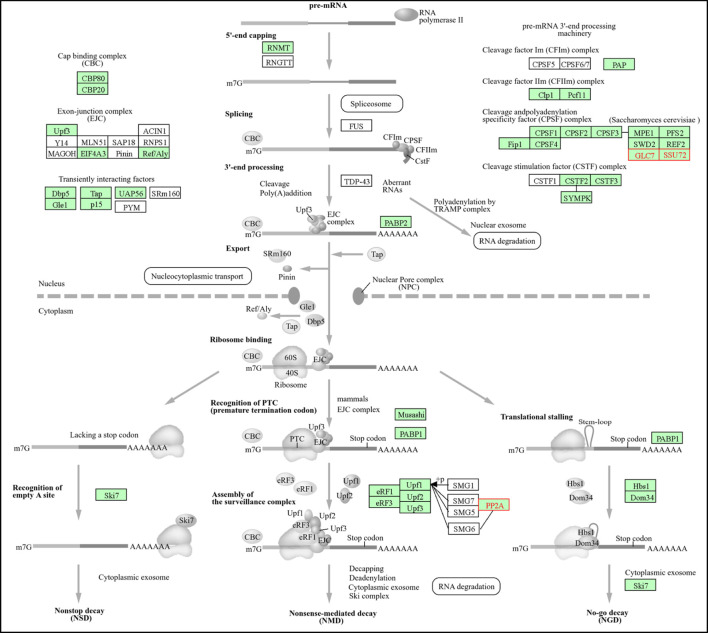
Shows the KEGG map of the mRNA surveillance pathway in *Saccharomyces cerevisiae* as reported by Kanehisa and Goto (2000). The pathway depicts the involvement of phosphatases (TPD3, PPH21, PPH22, GLC7, CDC55, SSU72, RTS1, PPQ1) in mRNA quality control, including nonsense-mediated decay, translation regulation, and coordination with transcription and cell cycle processes.

Whereas, as illustrated in (Figure 6), the gene YAL016W (TPD3) encodes the regulatory subunit A of the heterotrimeric protein phosphatase 2A (PP2A) complex, which functions as the structural scaffold that facilitates the interactions among the catalytic subunits, specifically Pph21 and Pph22, and the regulatory subunits, Cdc55 and Rts1. TPD3 is integral to the regulation of translation and protein biosynthesis, exerting an indirect influence on nonsense-mediated mRNA decay (NMD) by preserving translational fidelity (Schuller et al., 2004). Also, the genes YDL134C (PPH21) and its paralog YDL188C (PPH22) encode the catalytic subunits of protein phosphatase 2A (PP2A). PPH21 plays a crucial role in the modulation of mRNA stability, the regulation of cell cycle checkpoints, and the translation process under stress conditions. In contrast, PPH22, despite being functionally redundant with PPH21, is specifically activated under DNA replication stress conditions, thereby establishing a connection between genomic stability and mRNA quality control mechanisms. Moreover, both catalytic subunits are essential for the orchestration of the dephosphorylation of checkpoint proteins, which are critical for the synchronization of cell cycle progression with mRNA metabolic activities (Haluska et al., 2021; Shen et al., 2025). Furthermore, the gene YER133W, also known as GLC7, encodes a Type 1 serine/threonine phosphatase that plays a critical role in the regulation of translation termination factors. This regulatory function is essential for the accurate recognition of nonsense codons and the subsequent activation of nonsense-mediated mRNA decay. Additionally, Glc7 exhibits interactions with cleavage and polyadenylation factors, thereby establishing a linkage between transcription termination and mRNA surveillance mechanisms (Guan et al., 2006). In a parallel manner, the gene YGL190C, referred to as CDC55, acts as a regulatory subunit B of PP2A. CDC55 specifically directs the activity of PP2A towards substrates involved in translation and RNA metabolism, thereby orchestrating the synchronization of cell cycle progression with mRNA quality control checkpoints and modulating the components of the Mitotic Exit Network (Baro et al., 2018; Philip et al., 2022). The gene YNL222W, also known as SSU72, encodes a C-terminal domain (CTD) phosphatase that is instrumental in the processing of mRNA 3′-end and transcription termination. This enzyme achieves its function by dephosphorylating the CTD of RNA Polymerase II, specifically targeting the Tyr-1 residues and possibly the Ser-5 residues. This activity serves as a nexus between transcriptional regulation and mRNA surveillance (Ganem et al., 2003). In parallel, YOR014W, denoted as RTS1, acts as an alternative regulatory B′ of protein phosphatase 2A (PP2A). RTS1 confers distinct substrate specificity compared to CDC55, facilitating the organization of septins during cytokinesis and contributing to DNA damage response mechanisms. This action further links genome integrity maintenance with mRNA quality control (Aouida et al., 2019). Additionally, the gene YPL179W, identified as PPQ1, encodes a phosphatase related to protein phosphatase 1 (PP1). PPQ1 is postulated to function within protein quality control pathways that are complementary to the activity of Glc7, thereby aiding in the integrated regulation of both mRNA surveillance and proteostasis.

## 6 Determination techniques and analytical methods for fungal phosphatases

### 6.1 Traditional colorimetric assays

The p-nitrophenyl phosphate (pNPP) assay is an established colorimetric technique that quantitatively evaluates enzymatic activity by measuring the hydrolysis of phosphomonoesters, which releases p-nitrophenolate. This liberated compound forms a yellow chromophore, detectable via spectrophotometry at an approximate wavelength of 405 nm (Rombola et al., 2014). This assay is widely applied in assessing the activities of alkaline and acid phosphatases, provided that appropriate buffering and pH adjustments are maintained. It applies to purified enzymes, cellular extracts, and environmental samples, contingent on the implementation of controls to account for background absorbance (Rombola et al., 2014). The utilization of pNPP, as a small, non-physiological substrate, provides rapid and high-throughput results; however, it may not differentiate between monoesterase and diesterase activities and may be influenced by sample components such as metal ions and inhibitors. These factors can alter extinction coefficients or enzyme kinetics, necessitating substrate controls and calibration curves to accurately compute kinetic parameters, including Km and Vmax (Rombola et al., 2014). The rate of enzymatic activity is critically dependent on pH and the substrate employed. Acid phosphatases and alkaline phosphatases have distinct pH optima, requiring different assay buffers to achieve maximal activity measurements (Della Monica et al., 2018; Freitas-Mesquita et al., 2025). Assays should be tailored to the specific enzyme class being studied (e.g., acidic conditions for acid phosphatases, alkaline conditions for alkaline phosphatases) and consider the use of multiple substrates (pNPP for general monoesterase activity; bis-pNPP or glycerophosphocholine for diesterase fractions) to explore substrate specificity, as evidenced by fractionation studies in *Aspergillus niger* and other fungi (Zyla and Gogol, 2002).

### 6.2 Fluorometric and luminescent techniques

Sensitivity and spatial resolution, ELF-97 phosphate (ELFP) serves as a fluorogenic substrate that, upon dephosphorylation, forms an insoluble fluorescent precipitate at the enzymatic site. This property facilitates high-resolution localization of extracellular or surface-bound phosphatase activity via microscopy or flow cytometry (Alvarez et al., 2003; Paragas et al., 2002). ELF-97 has been effectively employed to map ectomycorrhizal acid phosphatase activity within fungal tissues and to quantify cell-surface acid phosphatase (AP) activity in both planktonic and benthic systems, thereby demonstrating its utility in fungal and symbiotic contexts (Steenbergh et al., 2011). Kinetics and quantitative application: ELFP can be quantitatively analyzed when factors such as precipitation kinetics, substrate diffusion, and imaging conditions are precisely calibrated. Studies within plankton and microfluidics have shown that ELFP kinetics encompass extensive dynamic ranges and are capable of revealing single-cell level variations in AP expression thresholds (Girault et al., 2018; Meseck et al., 2009). The integration of confocal fluorescence imaging with ELFP, in conjunction with image analysis software such as ImageJ, facilitates the quantification of surface-localized enzyme activity within fungal structures (Štraus et al., 2017). The Methylumbelliferyl Phosphate (MUF-P) Assay: Underlying Principle and Benefits: Methylumbelliferyl Phosphate (4-methylumbelliferyl phosphate) functions as a soluble fluorogenic monoester substrate that liberates the fluorescent compound 4-methylumbelliferone (MUF) through hydrolysis. Compared to colorimetric assays, MUF assays exhibit enhanced sensitivity and are conveniently adaptable to microplate-based kinetic analyses, as well as to samples with low abundance (Cao et al., 2007; Girault et al., 2018). MUF-P is extensively utilized in environmental enzymatic assays and laboratory enzyme kinetics to determine maximum reaction velocity (Vmax) and the Michaelis constant (Km) for alkaline phosphatase activities in extracts or filtrates. Its utility is particularly pronounced in scenarios where low enzymatic activity or small sample volumes make colorimetric detection impractical (Cao et al., 2007).

### 6.3 Microscopic and imaging methods

Confocal laser scanning microscopy (CLSM), when utilized in conjunction with ELF-97 staining, facilitates the spatial mapping of phosphatase activity within fungal structures such as the mantle, Hartig net, extraradical mycelium, and mycorrhizal interfaces, thereby providing direct visualization of enzyme localization relative to host tissues (Alvarez et al., 2003; Dreyer et al., 2008). Additionally, two-photon or multiphoton imaging methodologies have been employed to assess enzyme activity within polymer or tissue matrices, enabling three-dimensional mapping of phosphatase distribution at subcellular resolution (Basu and Campagnola, 2004). Regarding image analysis, quantitative image processing (e.g., using ImageJ) allows for the measurement of signal intensity, area, and relative activity across tissue compartments. Published protocols outline the acquisition and post-processing steps for ELF-97 images in ectomycorrhizae and other systems to achieve reproducible localization and semi-quantitative comparisons (Paragas et al., 2002; Štraus et al., 2017). *In vivo* localization and visualization techniques involving histochemical localization with stains such as NBT/BCIP, X-Phos, and ELF-97 reveal *in situ* phosphatase activity and have localized acid phosphatases to fungal cytoplasm, cell walls, and interface zones in arbusculated roots and ectomycorrhizal tissues, indicating roles in phosphorus efflux or mobilization at symbiotic interfaces (Dreyer et al., 2008; Lemoine et al., 1992). These methodologies provide spatially resolved evidence of fungal phosphatase deployment in ecological contexts (Alvarez et al., 2003). However, limitations exist, as precipitating substrates like ELF-97 can impede downstream molecular analyses (e.g., protein extraction) and necessitate meticulous controls to prevent photobleaching and non-enzymatic deposition. Therefore, complementary biochemical quantification methods (e.g., MUF or pNPP assays) are recommended to validate imaging results (Girault et al., 2018; Paragas et al., 2002).

### 6.4 Genomic and transcriptomic approaches

The cloning of fungal phosphatase genes and subsequent heterologous expression in various host systems, such as *Escherichia coli*, *Pichia pastoris*, and *Aspergillus* species, facilitates the biochemical characterization of recombinant enzymes. This approach also enables mutational analysis of active-site residues and the production of material for kinetic and structural studies. Gene cloning of phosphodiesterases and phosphatases in both fungal pathogens and model fungi has provided significant insights into substrate specificity and functional roles in cellular differentiation (Ramanujam and Naqvi, 2010). For instance, phosphodiesterase cloning in *Delftia* and studies on fungal phosphodiesterases have demonstrated the feasibility of heterologous expression for conducting kinetic and functional assays. Additionally, cloning of the PHO/AP gene in fungi has advanced the characterization of secreted acid phosphatases and members of the PAP family (Bernard et al., 2002; Ramanujam and Naqvi, 2010). Further Transcriptomic profiling using RNA-Seq under conditions of phosphate sufficiency versus deprivation, as well as varying carbon sources and stressors, elucidates the regulation of phosphatase genes and associated co-expressed networks, including interactions within the PHO regulon, TOR, and MAPK pathways. Studies conducted on plant-fungal interactions and fungal genomic surveys indicate a robust induction of PAP/AcPase transcripts under phosphate-starvation conditions, although transcriptional alterations may surpass enzymatic activity increases due to post-transcriptional regulatory mechanisms (Deng et al., 2020; Gomez-Gallego et al., 2025). Integrating RNA-Seq data with enzyme assays like MUF, pNPP, and ELFP imaging, alongside proteomics, offers a comprehensive view of regulatory mechanisms. This approach aids in identifying potential transcription factors and post-translational modifications that influence the deployment of phosphatases (Gomez-Gallego et al., 2025; Hurley et al., 2010). Proteomics and phosphoproteomics provide substantial protein-level evidence. Techniques such as shotgun proteomics or targeted mass spectrometry (MS), including selected reaction monitoring (SRM) and parallel reaction monitoring (PRM), are proficient in detecting and quantifying phosphatase proteins within secretomes, cell walls, and intracellular fractions. These methodologies corroborate the translation and secretion processes as predicted by transcriptomics analyses. Phosphoproteomics plays a crucial role by elucidating the substrates and signaling nodes modulated by protein phosphatases, indirectly demonstrated through the documentation of phosphorylation modifications following phosphatase perturbation (Huang et al., 2025). Functional integration is achieved by combining proteomic detection with activity assays, such as zymography and methylumbelliferyl phosphate (MUF) kinetics. This approach establishes a linkage between protein identity and catalytic functionality, thus enabling the validation of candidate phosphatases potentially responsible for the environmental activities observed (Bernard et al., 2002; Huang et al., 2025).

### 6.5 High-throughput and field-based methods

The utilization of microplate assays, particularly in the context of throughput and standardization, offers significant advantages for parallel processing of numerous samples using formats designed for pNPP, MUF-P, and other chromogenic or fluorogenic substrates. These formats are instrumental in condition screening, kinetic analyses, inhibitor testing, and conducting environmental surveys, with microplate ELF and MUF assays becoming pivotal in microbial and enzyme ecology research (Cao et al., 2007; Girault et al., 2018). Microplate assays facilitate the precise determination of enzymatic parameters such as Vmax and Km, as well as inhibitor responses across various replicates and experimental conditions, while utilizing minimal sample volumes (Girault et al., 2018). The automation and data handling aspects of these assays are enhanced through integration with plate readers, robotic systems, and standardized protocols, which collectively enhance reproducibility and comparability. However, this integration necessitates meticulous calibration across different instruments and stringent control for background and matrix effects. In the realm of portable and laboratory-independent devices, field applicability is significantly enhanced by the development of portable fluorometers and microfluidic devices that employ ELF or MUF substrates. These devices offer near-real-time detection of alkaline phosphatase activity in environmental samples such as plankton and hold considerable potential for *in situ* monitoring of fungal phosphatase activity within soils or rhizosphere microcosms (Wisuthiphaet et al., 2019). Furthermore, single-cell and microfluidic high-content platforms are capable of examining threshold responses of alkaline phosphatase expression to dissolved phosphate levels and can be adapted for use with fungal cell suspensions or microcolonies (Girault et al., 2018). However, the constraints with respect to field devices must be taken care of, and it must effectively overcome the effects of soil particulates, turbidity, and optical detection inhibitors like metals and humic acids. Subsequently, the sample pretreatment and introduction of matrix controls are vital to realize the attainment of credible field measurements. In a nutshell, the range of methods of detecting phosphatases is heterogeneous to represent the variety of applications and biological processes by which phosphatases are used, whether as part of isolated enzymatic kinetics or as biogeochemical cycling at the ecosystem scale, as summarized in (Table 3). Depending on the biological question, type of sample to be analyzed, level of analysis (molecular to field), sensitivity needs, and resource limitations are fundamental aspects of the choice of the method of analysis. These methodological strategies are not only indispensable to the biochemical characterization of fungal phosphatases under controlled laboratory conditions, but also, and most significantly, to the explanation and prediction of their roles within the complex ecological systems. Only the combined use of these analytical frameworks can allow us to rigorously assess the ecological importance of fungal phosphatases in terms of nutrient cycling, symbiotic relationships, and ecosystem resilience maintenance.

**TABLE 3:** Comparative analysis of principal methodologies for phosphatase detection.

Technique	Substrate	Principle	Sensitivity	Advantages	Limitations
pNPP Colorimetric	p-nitrophenyl phosphate	Yellow chromophore (p-nitrophenolate) released at a pH-dependent rate; absorbance measured at 405 nm	Low-moderate (mM range, Km typical)	High-throughput capable; widely standardized; inexpensive; long history of use; compatible with multiple enzyme classes	Cannot differentiate mono- vs. diesterase activity; matrix effects (metal ions, humic acids interfere); non-physiological substrate; requires background subtraction
MUF-P Fluorometric	4-methylumbelliferyl phosphate	Soluble fluorogenic substrate; releases fluorescent MUF (Ex 365 nm, Em 445 nm) upon hydrolysis	High (μM range Km achievable; 10–100× more sensitive than pNPP)	Superior sensitivity; adaptable to kinetic microplate readers; minimal sample volume required; suitable for environmental/low-activity contexts; good dynamic range	Matrix interference possible (though less than pNPP); fluorescence quenching by soil components; soluble substrate limits localization; photobleaching with extended incubation
ELF-97 (ELFP)	ELF-97 phosphate	Soluble, weakly fluorescent phosphorylated molecule; upon dephosphorylation forms bright yellow-green fluorescent precipitate (Ex 365 nm, Em 530 nm) at the enzymatic site	Very high (spatial localization at single-cell/organelle level; 500× more photostable than fluorescein)	Excellent spatial resolution; exceptional photostability (months to years persistence); multi-color compatible; minimal background autofluorescence; quantifiable via image analysis (ImageJ); non-soluble product remains at the site of activity	Precipitate can impede downstream molecular analyses (protein extraction); requires careful kinetics calibration; sample pretreatment is critical to prevent nonspecific deposition; not suitable for real-time kinetics; substrate diffusion-limited in dense tissues
Bis-pNPP/Glycerophosphocholine (GPC)	Bis-nitrophenyl phosphate or glycerophosphorylcholine	Synthetic or natural diester substrates; measure phosphodiesterase activity specifically	Moderate (substrate-dependent; GPC detection via phosphomolybdate assay is less sensitive)	Enables substrate specificity profiling; essential for distinguishing phosphomonoesterase vs. phosphodiesterase contributions; reflects ecological substrate types	Limited commercial availability for some substrates; slower reaction kinetics complicate high-throughput applications; GPC detection requires phosphomolybdate assay (separate step)
Zymography/Native PAGE Activity Staining	Substrate overlay (4-MUF, pNPP, or chromogenic) on native protein gels	Separation of isoenzymes by size/charge; in-gel substrate hydrolysis visualized as active bands	Moderate (detects active protein bands; isoenzyme-specific)	Reveals isoenzyme heterogeneity; provides approximate molecular weight estimates; combines separation with activity detection; amenable to mass spectrometric identification of active bands	Non-denaturing conditions may not resolve all isoforms; variable recovery of catalytic activity depending on protein stability; semi-quantitative only
Confocal Laser Scanning Microscopy (CLSM) + ELF-97	ELF-97 with optical sectioning	Combines spatial localization of ELF-97 signal with high-resolution confocal imaging (xy and z-axis); produces 3D reconstruction of activity distribution	Very high (subcellular/organellar resolution; 0.2–1 μm lateral resolution)	Unambiguous subcellular/tissue localization; 3D activity mapping; quantifiable intensity profiles; photostability allows extended scanning; can correlate activity with morphology	Photobleaching is possible despite ELF-97 photostability; optical penetration is limited to ∼100–200 μm; expensive instrumentation; expertise required for image acquisition and analysis; precipitate may obscure underlying structures
Two-Photon/Multiphoton Imaging	ELF-97 or fluorogenic substrates	Near-infrared excitation (700–900 nm) reduces photodamage and phototoxicity; enables imaging in thick tissue with minimal scattering	Very high (3D mapping at subcellular resolution in tissue depths up to 300–500 μm)	Superior optical penetration in scattering media (soil, tissue); reduced phototoxicity for live-cell imaging; minimal out-of-focus light; quantifiable 3D activity maps	Extremely high equipment and expertise costs; very limited accessibility; slow acquisition; not suitable for high-throughput work
Microplate Fluorometry (MUF-P or ELF-97)	MUF-P or fluorogenic substrates in 96-/384-/1536-well plates	Automated spectrofluorometry: reads fluorescence change over time; enables parallel processing of many samples	High (MUF-P typical sensitivity in μM range; fluorometric detection ∼100–1,000× plate reader sensitivity)	Standardized protocols; data output compatible with statistical analysis; minimal sample volume; automation possible; suitable for kinetic parameter determination; inhibitor screening	Instrument calibration is critical; matrix effects are still possible; requires positive/negative controls; pH and buffer system are critical for reproducibility
Droplet-Based Microfluidics	MUF-P, 3-O-methylfluorescein phosphate (OMFP), or chromogenic substrates in picoliter droplets	Miniaturized assay in ∼340 pL droplets generated at ∼40 Hz; fluorescence/absorbance measured per droplet; OMFP-based assays most common	Very high (sample volume ∼5,870,00× less than microplate; single-droplet resolution; enables cell-level analysis)	Massive reduction in sample/reagent volume; ultra-high-throughput (thousands of reactions/min); enables screening of phosphatase inhibitors; potential for single-cell enzymology; generates enormous datasets	Requires specialized expertise; expensive equipment; limited sample types (must be compatible with emulsion); results correlation with bulk assays needs validation; cell suspension preparation is critical
Flow Cytometry + ELF-97	ELF-97 phosphate substrate	Single-cell suspension; ELF-97 fluorescent precipitate detected as per-cell fluorescence intensity; enables population heterogeneity analysis	High (single-cell sensitivity; detects threshold responses to phosphate stress)	Enables detection of phosphatase expression heterogeneity; identifies threshold responses to nutrient signals; rapid population analysis (thousands of cells/sec); can combine with other markers (morphology, viability)	Requires single-cell suspension (challenging for filamentous fungi); fixation artifacts possible; limited spatial context; gating strategies must be optimized
Portable Fluorometer + ELF-97/MUF-P	ELF-97 or MUF-P in field-adapted format	Handheld fluorometer detects fluorescence from soil suspensions or filtrates; enables near-real-time field measurement	Moderate-high (field sensitivity ∼ micromolar range; less sensitive than laboratory fluorometers)	Field-portable; enables real-time monitoring without laboratory infrastructure; minimal sample preparation; rapid feedback for adaptive management	Matrix effects are severe (soil turbidity, metal ions, humic acids); requires extensive field pretreatment and calibration; limited optical path; less quantitative than laboratory methods
RNA-Seq/Transcriptomics	–	Quantifies phosphatase gene expression under various conditions (Pi replete vs. deplete, stress); integrates with enzyme assays for regulatory insight	–	Reveals phosphatase gene regulation; identifies upregulation of entire PHO/TOR regulons; discovers novel phosphatase family members; provides regulatory context for enzyme assays; enables co-expression network analysis	Does not measure protein abundance or activity (post-transcriptional regulation); requires RNA integrity; high technical variance possible; requires bioinformatic expertise; cost per sample moderate-high
Proteomics (MS)/Phosphoproteomics	–	Identifies phosphatase proteins in secretomes/cell walls/intracellular fractions; phosphoproteomics reveals phosphatase substrates (indirect activity evidence); combines with activity assays for validation	High (MS sensitivity down to femtomoles for target proteins; phosphoproteomics identifies specific phosphorylation sites modified by phosphatases)	Provides direct protein identification; enables mapping of phosphatase-substrate interactions; reveals post-translational modifications affecting activity; functional coupling of protein abundance with enzyme assays is possible	Does not directly measure enzyme activity; complex sample preparation; requires mass spectrometry expertise; limited throughput per cost; relative quantification prone to bias

## 7 Ecological functions of fungal phosphatases

### 7.1 Phosphorus mobilization in soil ecosystems

Fungal phosphomonoesterases, commonly referred to as acid phosphatases (PAPs), in conjunction with extracellular phosphodiesterases, facilitate the hydrolysis of organic phosphorus compounds, specifically phosphomonoesters and phosphodiesters. This enzymatic activity results in the liberation of inorganic orthophosphate, which becomes accessible to microbial communities and plant systems. Empirical evidence demonstrates that ericoid mycorrhizal fungi, cultivated with DNA serving as the exclusive phosphorus source, exhibit extracellular phosphomonoesterase and phosphodiesterase activities that support fungal biomass production and the utilization of organic phosphorus (Leake and Miles, 1996). Extensive surveys and enzymatic characterizations underscore the pivotal role of fungal extracellular phosphatases in the mineralization of organic phosphorus within soils and rhizospheres. These enzymes display activity profiles that are contingent upon fungal taxonomy and the chemical characteristics of phosphorus sources (Della Monica et al., 2018; Pandey et al., 2007). Specialized acid phosphatases such as phytases and PAPs advance the dephosphorylation of phytate and other inositol phosphates, processes that are pertinent to the degradation of feed/phytate and the turnover of soil organic phosphorus (Oh et al., 2004).

Fungi facilitate the mobilization of sparingly soluble inorganic phosphates through a suite of biochemical mechanisms. This process involves the secretion of organic acids, proton extrusion, and phosphatase activity, which collectively contribute to the solubilization and subsequent release of inorganic phosphate from mineral matrices, such as calcium, iron, and aluminum phosphates. Numerous studies involving isolation and culture have demonstrated that fungi belonging to genera such as *Aspergillus*, *Penicillium*, and *Trichoderma*, along with other soil fungi, are capable of solubilizing tricalcium phosphate, hydroxyapatite, and Al- and Fe-phosphates. These solubilization processes are frequently linked to the acidification of the medium by organic acids and the secretion of phosphatases and phytases that release phosphate from complexed forms (Barroso et al., 2006; Elias et al., 2016). For instance, isolates of *Aspergillus niger* have been shown to solubilize Ca- and Al-phosphates in media containing various carbon and nitrogen sources. This phenomenon is consistent with the hypothesis that solubilization driven by organic acids is complemented by enzymatic activity (Rinu and Pandey, 2010). Empirical evidence from field and applied studies supports the notion that PSF can enhance the availability of phosphorus for plants in soils, thereby holding potential as biofertilizers (Khuna et al., 2021; Qiao et al., 2019).

### 7.2 Mycorrhizal associations

The association with Arbuscular Mycorrhizal Fungi (AMF) facilitates enhanced P acquisition by host plants, primarily through the provision of mineralized Pi resulting from the enzymatic actions of the fungi, as well as the stimulation of soil phosphatase activity. Empirical evidence suggests that AMF colonization leads to an increase in phosphatase activity within the soil or rhizosphere and may induce the upregulation of both host and soil purple acid phosphatase (PAP)/acid phosphatase expression. This biochemical alteration potentially enhances the mineralization of organic phosphorus, thereby improving plant phosphorus uptake under conditions of phosphorus scarcity (Cao et al., 2022; Ren et al., 2018). Ultrastructural localization analyses have identified the presence of acid phosphatase at the arbuscular interface and within arbusculated coils. This observation implies a role for both fungal and plant phosphatases in the transfer of phosphorus across the symbiotic interface (Dreyer et al., 2008). Furthermore, genomic investigations have revealed the presence of conserved PHO/TOR and nutrient-signaling pathways within AM fungi, which align with the regulated deployment of phosphatases during symbiotic interactions (Zhou et al., 2021). Ectomycorrhizal fungi (EMF) synthesize both surface-bound and secreted phosphatases, facilitating the mobilization of organic phosphorus sources within forest soils. Utilizing methods involving ELF-97 and confocal imaging, researchers have localized surface-bound phosphatase activity on EMF hyphae and mycorrhizae. Functional assays demonstrate the capability of EMF to utilize DNA as a sole phosphorus source due to their extracellular phosphodiesterase and monoesterase activities (Alvarez et al., 2003; Leake and Miles, 1996). These extracellular enzyme activities underscore the role of EMF as pivotal agents in accessing organic phosphorus pools, such as nucleic acids and phospholipids, within forest litter and mineral soils. Ericoid and orchid mycorrhizae have been subjects of experimental research, particularly concerning their ability to utilize polymeric organic phosphorus (such as DNA) through the activity of extracellular enzymes, including phosphodiesterase and phosphomonoesterase. This enzymatic activity is crucial in facilitating host nutrition within acidic and organic-rich soil environments, commonly found in heathlands and bogs (Leake and Miles, 1996). Similarly, orchid mycorrhizae and ericoid associations have been noted to exploit organic phosphorus pools through the secretion of acid phosphatases and phytases. This process aligns with symbiotic strategies that prioritize the mineralization of organic phosphorus over mineral phosphorus acquisition (Ezawa et al., 2005).

### 7.3 Plant–fungal nutrient exchange

The transfer of phosphate from fungi to plants is facilitated at symbiotic interfaces, characterized by fungal acquisition through phosphatases and transporters, followed by efflux and transfer to plant cells. The presence of acid phosphatase at the AM interface, along with the upregulation of host and fungal phosphatase genes during symbiosis, suggests the involvement of enzymatic mineralization and membrane transport in the phosphorus ion flow from fungi to plants (Ren et al., 2018). Moreover, fungal polyphosphate metabolism, mediated by polyphosphatases, affects intracellular Pi reservoirs, which can influence mobilization and transfer processes. Disruption in polyphosphate mobilization can alter Pho4 activation and fungal virulence in model organisms, indicating that pathways of phosphate storage and mobilization interact with phosphate signaling and transfer mechanisms (Ahmed et al., 2022). This effect is attributed to the fungi’s ability to mobilize phosphorus through mechanisms such as the secretion of organic acids, phosphatases, and phytases, thereby promoting measurable plant growth benefits (Qiao et al., 2019; Sane and Mehta, 2015). The observed advantages in plant growth have spurred the development of phosphate-solubilizing fungal (PSF) strains as biofertilizers aimed at achieving sustainable agricultural practices (Khuna et al., 2021).

## 8 Sustainable applications and biotechnological potential

### 8.1 Agricultural applications

The mechanistic foundation underlying plant-available phosphorus enhancement by phosphate-solubilizing fungi involves two primary processes: (i) the secretion of extracellular phosphomonoesterases, specifically acid phosphatases, and phosphodiesterases, which facilitate the mineralization of organic phosphorus, and (ii) the production of organic acids that solubilize otherwise insoluble mineral phosphates, including calcium, iron, and aluminum compounds. These processes collectively release orthophosphate, which is readily absorbed by plants (Arias et al., 2023; Sang et al., 2022). Empirical research has demonstrated that isolates belonging to genera such as *Aspergillus*, *Penicillium*, and *Mortierella* exhibit both acid phosphatase and phytase activities, along with acidifying metabolic processes that are crucial for PSF functionality (Arias et al., 2023; Jaskulska et al., 2020). The documented benefits of PSF inoculation include enhancements in plant phosphorus content and growth across both controlled and field environments. Notably, isolates of *Penicillium* and *Aspergillus* have been shown to promote growth in *Pinus massoniana*, *Arabidopsis*, onion, and various other crops under phosphorus-limited conditions, suggesting their potential utility as biofertilizers (Arias et al., 2023; Qiao et al., 2019). These studies have correlated measurable phosphatase and phytase activities, as well as organic acid secretion, with plant physiological responses, thereby substantiating the functional significance of fungal enzymes *in situ* (Qiao et al., 2019). The variability in efficacy and the factors influencing it are notable, with the field performance of fungal inoculants being context-dependent. Variables such as soil phosphorus status, indigenous microbial communities, pH, temperature, and organic matter content significantly affect PSF establishment and efficacy. It is observed that the addition of mineral phosphorus generally suppresses microbial phosphatase activity and diminishes PSF benefits, whereas organic amendments and low-phosphorus soils enhance PSF activity (Jaskulska et al., 2020; Shi et al., 2024). Strains of *Aspergillus* that are tolerant to cold and varying pH levels maintain solubilization activity under diverse conditions, illustrating strategic strain selection for deployment in marginal environments (Rinu et al., 2013). Co-inoculation and microbiome interactions: The co-inoculation of AMF and PSF or PSB, or the recruitment of beneficial bacterial partners, may enhance the mineralization of organic phosphorus and the uptake of phosphorus by plants through complementary mechanisms. AMF can stimulate associated bacteria that are capable of mineralizing phytate, while fungal exudates, such as sugars, can recruit phosphatase-active bacteria, thereby increasing overall phosphorus mobilization (Wang et al., 2017; Zhang et al., 2018). These synergistic interactions advocate for the development of multi-partner inoculants or microbiome management strategies to stabilize field performance. Compatibility with organic amendments: The application of organic fertilizers and residues enhances soil organic matter and stimulates soil phosphatase activities. Organic systems that rely less on mineral phosphorus fertilizers can benefit from PSF that mineralize organic phosphorus pools, thereby enhancing phosphorus resupply to plants (Shi et al., 2024). Empirical evidence indicates that organic and combined organic-mineral fertilization regimes sustain higher enzyme activities and phosphorus availability compared to mineral-only inputs, suggesting that the utilization of PSF can align with the objectives of organic management (Zhang et al., 2023). As summarized in (Table 4), representative examples of phosphate-solubilizing fungi and their beneficial effects on a wide range of host plant species are presented. The documented outcomes include enhanced phosphorus acquisition, improved biomass accumulation, increased yield, and the stimulation of plant growth regulators. The investigation of plant-soil feedback inoculation trials has elucidated crop-specific advantages, demonstrating that cereals and horticultural crops, such as maize, onions, and various vegetables, exhibit enhancements in yield and nutrient uptake following inoculation with mineral-solubilizing fungi. Conversely, arboreal seedlings, including species like Pinus and *Pinus massoniana*, show improved growth in phosphorus-deficient soils due to inoculation with specific strains of *Penicillium* and *Aspergillus* (Arias et al., 2023; Khuna et al., 2021; Qiao et al., 2019). In leguminous crops, which frequently associate with arbuscular mycorrhizal fungi and nitrogen-fixing symbionts, plant-soil feedback that augment phosphorus availability can enhance symbiotic nitrogen fixation. Nonetheless, the extent of these benefits is contingent upon the root architecture and mycorrhizal strategies inherent to each crop species (Liu et al., 2015; Nasto et al., 2017). To optimize outcomes and mitigate failure risks across varied agricultural systems, it is crucial to judiciously select fungal strains that possess optimal phosphatase activity and pH compatibility, coupled with the requisite stress tolerance, such as cold adaptation for temperate agroecosystems (Rinu et al., 2013; Sang et al., 2022).

**TABLE 4:** Host plants benefit from phosphate-solubilizing fungi and associated agricultural benefits.

Host plant	PSF species	Effects	References
Wheat	*P. bilaiae*, and *Penicillium* spp.	The number of leaves per plant rose; dry matter accumulation rose; grain weight and grain yield rose	Patil et al. (2012)
Wheat	*P. oxalicum*	Enhanced growth and productivity	Singh and Reddy (2011)
Maize and corn	*Aspergillus* sp., *Penicillium* sp., and *Cephalosporium* sp	Phosphorus uptake and plant dry mass rose	Kassim (2011)
Maize	*Pleurotus ostreatus*	Enhancement of root and shoot lengths, fresh and dry root mass, fresh and dry shoot mass, and chlorophyll concentration	Maharana et al. (2021)
Maize	*Mortierella capitata*	Enhanced biomass, chlorophyll, and gibberellic acid levels	Li et al. (2020)
Tomato	*A. awamori*, and *Trichoderma viride*	Enhanced yield	Sibi (2011)
Tomato	*Pochonia chlamydosporia*	Increase in the number of lateral roots and in the overall root biomass of seedlings	Zavala-Gonzalez et al. (2015)
*Amaranthus cruentus* L	*Aspergillus niger*	Leaf count rose; shoot dry biomass increased; overall phosphorus content went up	Reena et al. (2013)
Chickpea (*Cicer arietinum*)	*Trichoderma harzianum* and *A. niger*	Shoot length was enhanced; root length was enhanced; dry biomass of both shoot and root increased	Yadav et al. (2011)
Groundnut	*A. niger*, *P. notatum*	Yield improved; protein percentage rose; and oil content percentage increased	Manivannan et al. (2012)
Cucumber	*Aspergillus* sp	Enhanced plant growth, biomass accumulation, leaf area, and chlorophyll content	Islam et al. (2014)
Lettuce	*P. albidum*	Rise in total weight	Morales et al. (2011)
*Dalbergia sissoo*	*P. chrysogenum*	Improve biomass production	Sujata Dash et al. (2013)
*Avena sativa*	*Geomyces pannorum, and Paecilomyces carneus*	Enhance soil phosphorus availability and reduce populations of plant-parasitic nematodes	Lima-Rivera et al. (2016)
Banana	*Trichoderma*, and *Purpureocillium*	Actively produce indole-3-acetic acid (IAA) and convert insoluble phosphate into a soluble form	Napitupulu et al. (2021)
*Soy bean*	*Trichoderma*	Enhancement of plant growth leading to improved phosphorus uptake efficiency	Bononi et al. (2020)
Rapeseed	*Penicillium oxalicum*	Dissolve inorganic P and convert organic P into mineral forms	Wang et al. (2021)
Avocado	*Mortierella* sp	Enhancement of plant growth and phosphorus acquisition	Tamayo-Velez and Osorio (2017)

### 8.2 Industrial bioprocessing

The utilization of phytase, particularly fungal phytases such as histidine acid phytases and alkaline phytases, has been industrially applied to degrade phytate present in monogastric animal feeds. This process increases the bioavailability of phosphorus and decreases phosphorus excretion, thereby minimizing environmental pollution (Oh et al., 2004; Singh and Satyanarayana, 2010). Thermostable fungal phytases, such as those derived from *Sporotrichum thermophile* and various *Aspergillus* species, have demonstrated significant translational potential as fungal phosphatases (Singh and Satyanarayana, 2010). The application of feed phytase presents an environmental advantage by reducing the necessity for inorganic phosphorus supplements and thereby decreasing phosphorus content in manure, which mitigates eutrophication risks commonly associated with livestock production. In the realm of commercial enzyme supply, fungal extracellular phosphatases and phytases are well-suited for industrial fermentation processes, followed by downstream purification and formulation. These enzymes are utilized in a variety of applications, including detergents, dephosphorylation reactions, and bioremediation. The substantial secretory capacity of filamentous fungi, such as *Aspergillus* and *Trichoderma*, facilitates the scalable production of these enzymes (Brown et al., 2013). Through enzyme engineering and process optimization, the activity, stability, and substrate range of these enzymes can be significantly enhanced for industrial applications. The concept of resource valorization involves integrating phosphatase-producing fungi into biorefineries, which could improve the recovery of phosphorus from agricultural residues and processing wastes, such as oilseed meals and brewers’ wastes, through enzymatic dephosphorylation before their downstream utilization or nutrient recycling. This integration supports the establishment of circular nutrient flows (Chen et al., 2025; Zyla et al., 1995). However, it necessitates a comprehensive techno-economic assessment and optimization of enzyme activity within complex biomass streams.

Further fungi play a significant role in the stabilization of soil organic carbon through the production of persistent fungal necromass and the formation of soil aggregates. By enhancing soil structure and facilitating the binding of organic matter, fungal biomass exerts an indirect influence on phosphorus retention and the pools of organic P that are available for mineralization (Rashid et al., 2016). Management practices that increase fungal biomass, such as the promotion of mycorrhizal associations and the reduction of tillage, can provide dual benefits for both carbon sequestration and phosphorus cycling. Stoichiometric constraints: The availability of phosphorus limits primary productivity and carbon sequestration at the ecosystem level; biological mobilization of phosphorus by fungal phosphatases influences plant growth and carbon inputs to soils, thereby creating feedback mechanisms between the phosphorus and carbon cycles (Liu et al., 2014). Climate-induced changes, including warming and altered precipitation patterns, have the potential to modify microbial phosphorus utilization and sorption dynamics, which may reduce biological phosphorus availability despite an increase in turnover. This underscores the necessity of integrating fungal phosphorus processes into climate-smart soil management strategies (Tian et al., 2023).

## 9 Conclusion and future research directions

Fungal phosphatases constitute key biochemical mediators at the interface of intracellular signal transduction, nutrient metabolism, and the global biogeochemical cycling of phosphorus. The fungal phosphatase repertoire is highly diverse and encompasses phosphomonoesterases (including acid and alkaline phosphatases, purple acid phosphatases, and phytases), phosphodiesterases, polyphosphatases, as well as protein dephosphorylating enzymes such as serine/threonine- and tyrosine-specific phosphatases. Comparative genomic and phylogenetic investigations reveal pronounced sequence divergence and lineage-specific gene family expansions among fungal clades, indicative of substantial structural and regulatory heterogeneity that underlies fungal ecological plasticity and phosphorus acquisition strategies. Fungi occupy a central position in the biotransformation of both organic and inorganic phosphorus forms. By secreting extracellular phosphomonoesterases and phosphodiesterases, fungal communities mineralize a broad spectrum of organic phosphorus substrates, including phytate, nucleic acids, and phospholipids, thereby liberating orthophosphate for subsequent assimilation by fungi and plants. Enzymes with high substrate specificity, particularly phytases, play a pivotal role in phytate depolymerization, with direct consequences for soil phosphorus availability, the development of biofertilizers, and the efficiency of phosphorus utilization in animal nutrition. In parallel, numerous fungi facilitate the mobilization of mineral-associated phosphate pools (e.g., calcium-, iron-, and aluminium-bound phosphates) through the secretion of organic acids and the modulation of rhizosphere pH, processes that collectively enhance soil productivity and nutrient bioavailability. The expression and catalytic activity of fungal phosphatases are stringently controlled by interconnected signaling and regulatory networks, including the target of rapamycin, calcineurin, and mitogen-activated protein kinase pathways, together with carbon nutrient-sensing systems. These regulatory circuits integrate phosphatase function with cellular energy status, nutrient supply, and abiotic and biotic stress responses, thereby enabling fungi to dynamically optimize phosphorus acquisition under temporally and spatially variable environmental conditions.

Recent advances in analytical methodologies such as refined colorimetric and fluorometric assays, zymography, mass spectrometry-based proteomics, and high-resolution microscopy have substantially improved both the quantitative and spatial characterization of phosphatase activity. These technologies facilitate the discrimination of individual isozymes, elucidate their localization and function *in situ*, and enhance the reproducibility and comparability of experimental data across studies. From ecological and agronomic perspectives, mycorrhizal and saprotrophic fungi represent major drivers of terrestrial phosphorus cycling. A substantial body of evidence indicates that phosphate-solubilizing and phytase-secreting fungi can increase phosphorus use efficiency, promote seedling establishment, and augment crop yields. Nevertheless, the performance of fungal inoculants in field conditions is highly context-dependent, being strongly influenced by soil physicochemical properties, plant genotype, resident microbiomes, and climatic factors. This variability underscores the necessity of site-specific validation and optimization across distinct edaphic and agroclimatic settings. When appropriately integrated into management practices, enhanced fungal-mediated phosphorus mobilization may permit reductions in inorganic phosphorus fertilizer inputs by approximately 20%–40% in certain production systems, while concurrently mitigating phosphorus losses via runoff and reducing the risk of eutrophication. Furthermore, extensive fungal hyphal networks contribute to the long-term stability and functioning of ecosystems by promoting soil aggregate formation, stabilizing soil organic matter, and facilitating carbon sequestration.

Despite substantial progress, several critical knowledge gaps persist that constrain the reliable, scalable, and field-relevant application of fungal phosphatases. Future research should prioritize: (i) multi-location and multi-crop field trials across a broad spectrum of soil types (pH 4.5–8.5) to translate laboratory-scale efficacy into quantifiable agronomic outcomes and to realistically evaluate the potential for synthetic fertilizer substitution; (ii) integrated multi-omics investigations (transcriptomics, proteomics, and metabolomics), coupled with quantitative measurements of phosphatase activity and plant nutrient uptake, to elucidate mechanistic links between molecular regulatory networks and phosphorus mobilization under environmental conditions; (iii) comparative genomics combined with functional validation, including CRISPR-based engineering of phosphatase-producing fungal strains, to concomitantly enhance multiple phosphatase systems and introduce broad-spectrum stress-tolerance traits; and (iv) international harmonization of phosphatase assay methodologies through standardized protocols, inter-laboratory proficiency testing, and the development of field-portable diagnostic platforms, thereby enabling regulatory implementation and robust cross-study comparability. Addressing these research priorities will be crucial for transitioning fungal phosphatase research from predominantly mechanistic insights toward dependable, scalable, and sustainable phosphorus management strategies in agricultural systems.
